# The Fission Yeast Stress-Responsive MAPK Pathway Promotes Meiosis via the Phosphorylation of Pol II CTD in Response to Environmental and Feedback Cues

**DOI:** 10.1371/journal.pgen.1002387

**Published:** 2011-12-01

**Authors:** Yuko Sukegawa, Akira Yamashita, Masayuki Yamamoto

**Affiliations:** Department of Biophysics and Biochemistry, Graduate School of Science, University of Tokyo, Tokyo, Japan; National Cancer Institute, United States of America

## Abstract

The RRM-type RNA-binding protein Mei2 is a master regulator of meiosis in fission yeast, in which it stabilizes meiosis-specific mRNAs by blocking their destruction. Artificial activation of Mei2 can provoke the entire meiotic process, and it is suspected that Mei2 may do more than the stabilization of meiosis-specific mRNAs. In our current study using a new screening system, we show that Mei2 genetically interacts with subunits of CTDK-I, which phosphorylates serine-2 residues on the C-terminal domain of RNA polymerase II (Pol II CTD). Phosphorylation of CTD Ser-2 is essential to enable the robust transcription of *ste11*, which encodes an HMG-type transcription factor that regulates the expression of *mei2* and other genes necessary for sexual development. CTD Ser-2 phosphorylation increases under nitrogen starvation, and the stress-responsive MAP kinase pathway, mediated by Wis1 MAPKK and Sty1 MAPK, is critical for this stress response. Sty1 phosphorylates Lsk1, the catalytic subunit of CTDK-I. Furthermore, a feedback loop stemming from activated Mei2 to Win1 and Wis4 MAPKKKs operates in this pathway and eventually enhances CTD Ser-2 phosphorylation and *ste11* transcription. Hence, in addition to starting meiosis, Mei2 functions to reinforce the commitment to it, once cells have entered this process. This study also demonstrates clearly that the stress-responsive MAP kinase pathway can modulates gene expression through phosphorylation of Pol II CTD.

## Author Summary

Hundreds of genes are newly expressed during meiosis, a process to form gametes, and the control of meiosis-specific gene expression is not simple. The master regulator of meiosis in fission yeast, Mei2, blocks an RNA destruction system that selectively degrades meiosis-specific mRNAs, highlighting the importance of post-transcriptional control in meiotic gene expression. Here we present another example of unforeseen regulation for meiosis. Ste11 is a key transcription factor responsible for the early meiotic gene expression in fission yeast. The *ste11* gene is transcribed robustly only when serine-2 residues on the C-terminal domain (CTD Ser-2) of RNA polymerase II are phosphorylated. We show that the stress-responsive MAP kinase cascade transmits the environmental signal to stimulate CTD Ser-2 phosphorylation. Sty1 MAP kinase appears to phosphorylate and activate the catalytic subunit of CTDK-I, which in turn phosphorylates CTD Ser-2. We demonstrate further that Mei2, expression of which depends on Ste11, can activate the MAP kinase cascade, forming a feedback loop. Thus, we clarify here three important issues in cellular development: the physiological role of CTD Ser-2 phosphorylation, the molecular function of the stress-responsive MAP kinase pathway, and the presence of positive feedback that reinforces the commitment to meiosis.

## Introduction

The cell cycle programs for mitosis and meiosis appear to be strictly segregated from each other, although they are likely to have molecular mechanisms in common. Analyses in lower eukaryotes have shown that factors required exclusively for meiosis, generated through the transcriptional activation of meiosis-specific genes, are largely responsible for the segregation of these two processes [1], [2]. In addition, we have reported previously in fission yeast that meiosis-specific mRNAs transcribed at the wrong time during the mitotic cell cycle are removed selectively by nuclear exosomes, thereby preventing the inappropriate expression of the meiotic program in mitotic cells [3], [4]. The master meiotic regulator in fission yeast, Mei2, an RNA-binding protein with three RRM domains [5]–[7], suppresses the function of this selective removal system by sequestering a key component Mmi1, which is an RNA-binding protein of the YTH family [3]. Mei2 thus ensures full expression of meiosis-specific genes and facilitates execution of the meiotic program (reviewed in [8]). However, it is unlikely that the function of Mei2 in meiosis is confined to the tethering of Mmi1 as the artificial inactivation of Mmi1 does not induce the full meiotic program, whereas the experimental induction of the activated form of Mei2 does so [3], [6]. The mechanisms and pathways by which Mei2 promotes the entire meiotic program is therefore a subject of considerable interest.

To identify possible upstream or downstream effectors of Mei2, we devised a new screening system and found that a subunit of CTDK-I, which is a CDK-like kinase complex that phosphorylates the C-terminal repeat domain of the largest subunit of RNA polymerase II (Pol II CTD) [9], [10], could genetically interact with Mei2. More specifically, the phosphorylation of Pol II CTD by CTDK-I was found to affect the expression of *ste11*, which encodes a key transcription factor that regulates the *mei2* gene. Pol II CTD serves as a binding scaffold for a variety of nuclear factors, and its phosphorylation status has been implicated in regulation of an ever-increasing number of functions necessary to execute complex transcriptional processes [9], [10]. Our aforementioned findings indicate that the phosphorylation of Ser-2 residues on Pol II CTD in fission yeast is unique in that it is required mainly for the meiotic program, via the activation of *ste11* transcription, but is not absolutely necessary for the mitotic program. Essentially the same conclusions have been reached independently by others, through global gene expression analysis [11]. Here we further show that the stress-responsive MAP kinase cascade is crucial for the phosphorylation of Ser-2 residues under nutrient starvation, which is a condition suitable for meiosis. We also show that artificially activated Mei2 has the potential to promote the phosphorylation of Ser-2 residues on Pol II CTD via the stress-responsive MAP kinase cascade, irrespective of the nutrient conditions.

Taken together, the results of our present study demonstrate a new regulatory paradigm for meiosis by Mei2 in fission yeast, i.e., that this master meiotic regulator ensures the commitment to meiosis by strengthening the transcription of *ste11* via a feedback loop comprising the stress-responsive MAP kinase cascade and the phosphorylation of Pol II CTD by CTDK-I.

## Results

### Isolation of *lsg1* as a suppressor of the ectopic meiosis induced by the artificial activation of the meiotic regulator Mei2

The haploid fission yeast strain JV312 harbors the *mei2-L-SATA* allele driven by the authentic *mei2* promoter. This allele contains a combination of two mutations, *mei2-L* and *mei2-SATA*. The former mutation confers temperature-sensitivity to the Mei2 protein (our unpublished results), whereas the latter activates this gene constitutively, overriding the inhibitory phosphorylation by Pat1 kinase [6]. JV312 cells arrest during vegetative growth and induce ectopic meiosis at 25°C because the Mei2-L-SATA protein is functional at this temperature. However, these cells continue vegetative growth at 32°C because Mei2-L-SATA is then inert and does not interfere with cell growth pathways. To identify novel upstream regulators or downstream effectors of Mei2, we screened for suppressor mutants that could grow at 25°C by insertional mutagenesis of JV312 (see Materials and Methods). Several suppressor mutants were thereby isolated, one of which was found to contain an insertion in SPBC4B3.08, which is annotated in the fission yeast database (http://old.genedb.org/genedb/pombe/) to encode a homologue of the γ subunit of RNA polymerase II C-terminal domain kinase I (CTDK-I). CTDK-I belongs to the CDK family, but in addition to the catalytic subunit α and the cyclin-like regulatory subunit β conserved among these family members, it contains a third γ subunit [12], [13]. In fission yeast, the *lsk1* and *lsc1* genes encode the α and β subunits of the CDK proteins, respectively [14], [15]. Hereafter, we designate SPBC4B3.08 as *lsg1*.

**Figure 1:**
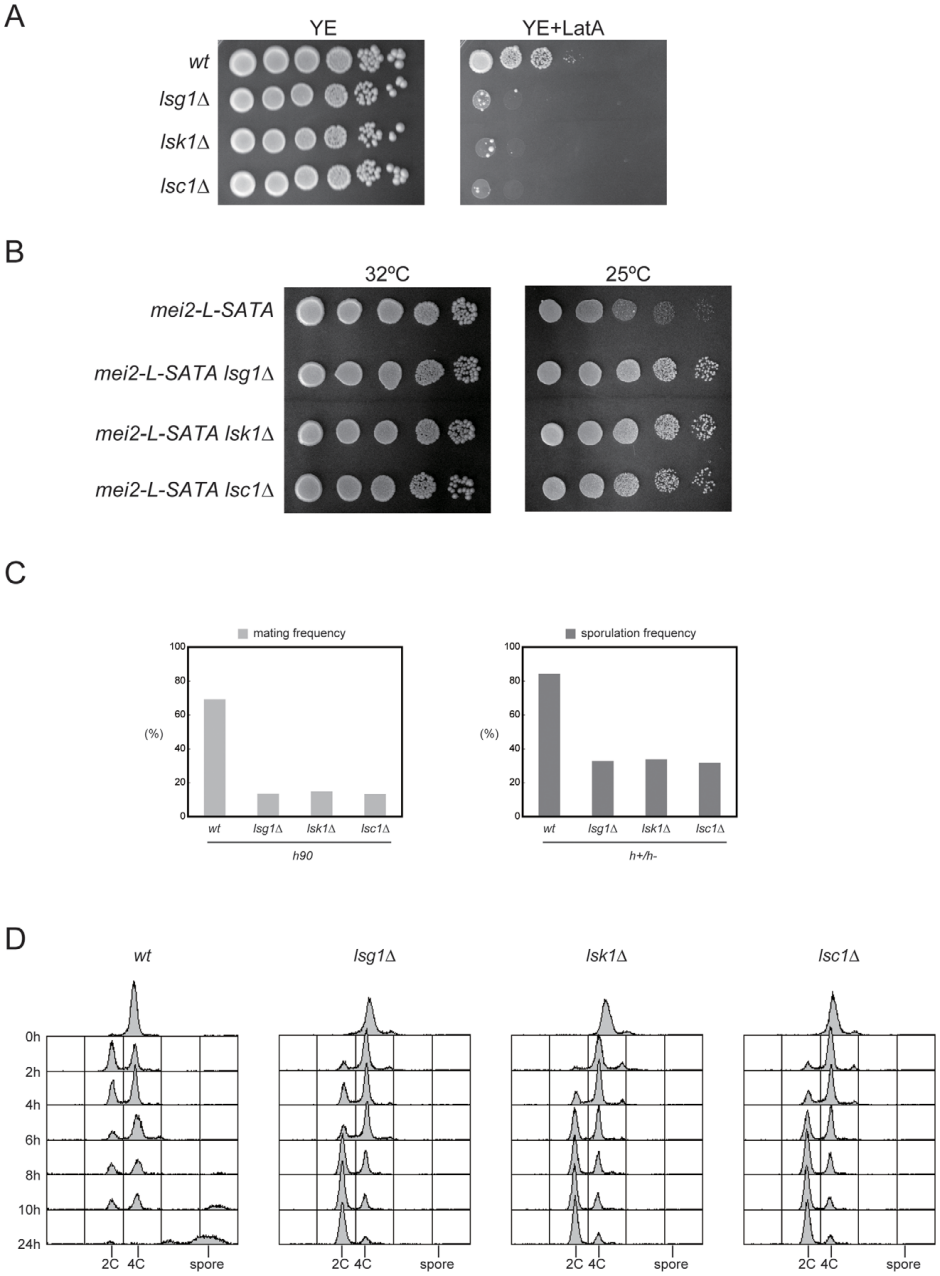
Phenotypes of the mutants defective in each CTDK-I subunit. (A) Sensitivity to Latrunculin A. Growth of haploid strains JY450 (wild-type), JT659 (*lsg1Δ*), JT660 (*lsk1Δ*) and JT661 (*lsc1Δ*) was examined on YE plates with or without addition of 0.5 µM Latrunculin A. Ten-fold serial dilutions of each strain were spotted and incubated at 30°C for four days. (B) Suppression of *mei2-L-SATA* by *lsg1Δ*, *lsk1Δ*, or *lsc1Δ*. Ten-fold serial dilutions of haploid strains JV312 (*mei2-L-SATA*), JT662 (*mei2-L-SATA lsg1Δ*), JT663 (*mei2-L-SATA lsk1Δ*), and JT664 (*mei2-L-SATA lsc1Δ*) were spotted onto SD plates and incubated either at 32°C or 25°C for four days. (C) Reduced mating and sporulation frequencies in the CTDK-I deletion mutants. Cells of the homothallic (*h* ^90^) haploid strains JY450 (wild-type), JT659 (*lsg1Δ*), JT660 (*lsk1Δ*), and JT661 (*lsc1Δ*) were examined microscopically for their conjugation frequency after incubation on SSA plates at 30°C for three days (left panel). Cells of heterozygous diploid (*h* ^+^/*h* ^−^) strains JY362 (wild-type), JT665 (*lsg1Δ*/*lsg1Δ*), JT666 (*lsk1Δ*/*lsk1Δ*), and JT667 (*lsc1Δ*/*lsc1Δ*) were examined microscopically for their sporulation frequency after incubation on SSA plates at 30°C for two days (right panel). (D) DNA content of the diploid strains JY362, JT665, JT666, and JT667 exposed to nitrogen starvation. Cells were cultured in liquid MM medium to mid-log phase and then shifted to MM-N medium. Aliquots were taken at the indicated intervals and the cellular DNA content was determined by FACS analysis.

Because the level of homology between fission yeast Lsg1 and *Saccharomyces cerevisiae* CTDK-I γ (CTK3) was found not to be high (a 24% amino acid identity; Figure S1), we examined whether Lsg1 was indeed a functional homolog of CTDK-I γ. We constructed the *lsg1*-deletion strain by replacing the entire *lsg1* ORF with a drug-resistant cassette, and compared its phenotype with that of *lsk1Δ* and *lsc1Δ*. The *lsg1Δ* strain exhibited no significant defects in mitotic growth, like the *lsk1Δ* and *lsc1Δ* strains previously analyzed [14], [15] (Figure 1A). The doubling time in liquid YE medium at 30°C was 2.1 h for the wild-type, 2.2 h for *lsg1Δ*, 2.3 h for *lsk1Δ*, and 2.2 h for *lsc1Δ*, respectively. However, *lsg1Δ* cells showed hypersensitivity to Latrunculin A, an inhibitor of actin polymerization, which was a phenotype reported previously for *lsk1Δ* and *lsc1Δ* [14], [15] (Figure 1A). In addition, both *lsk1Δ* and *lsc1Δ* could suppress the growth defect of *mei2-L-SATA* at 25°C as efficiently as *lsg1Δ* (Figure 1B). These observations confirmed that *lsg1* indeed encodes the CTDK-I γ subunit, and indicated that loss of CTDK-I activity is responsible for the suppression of *mei2-L-SATA*.

### Deletion mutants of the CTDK-I subunits are defective in sexual development

Although deletion of the gene encoding each CTDK-I subunit led to no obvious defect under normal growth conditions, these deletion mutants all showed impairments in conjugation and sporulation under starved conditions. Under these conditions, haploid *lsg1Δ*, *lsk1Δ* or *lsc1Δ* cells conjugated at a lower frequency than wild-type cells, and diploid *lsg1Δ*, *lsk1Δ* or *lsc1Δ* cells underwent azygotic meiosis and sporulation at a lower frequency than wild-type cells (Figure 1C). We further found that the progression of the meiotic cell cycle was significantly retarded in the CTDK-I subunit mutants. Fluorescence-activated cell sorting (FACS) analysis indicated that diploid *lsg1Δ*, *lsk1Δ* or *lsc1Δ* cells began to arrest in G1 phase as late as eight hours after the shift to nitrogen starvation and showed minimal premeiotic DNA synthesis even after 24 hours. In contrast, wild-type cells began to arrest in G1 phase after two hours and completed premeiotic DNA synthesis at between 2 and 6 hours (Figure 1D).

### The loss of ste11 expression is the major cause of the mating and sporulation deficiency in the CTDK-I mutants

Our observations that the CTDK-I deletion mutants were defective in sexual development and could suppress growth deficiency, evoked by the *mei2-L-SATA* allele, led us to speculate that the expression of *ste11*, which encodes an HMG-family transcription factor, might be impaired in these mutants. Our reasoning was that 1) Ste11 regulates the transcription of many genes essential for sexual development, including *mei2* [16]; 2) the deletion of *ste11* has been shown to suppress ectopic meiosis induced by the *pat1* mutation and restore vegetative growth, by blocking the expression of *mei2* [17], [18]; and 3) we had noticed that *ste11Δ* cells show G1 arrest retardation under conditions of nitrogen starvation, even more extensively than *lsg1Δ*, *lsk1Δ* or *lsc1Δ* cells, while *mei2Δ* cells are not so much affected (Figure S2A). We thus analyzed the transcription of *ste11* in *lsg1Δ* cells and found that it was significantly suppressed (Figure S2B). Because requirement of CTDK-I for the expression of *ste11* has been independently discovered and already reported by Hermand and his colleagues [11], we briefly summarize our data that confirm their conclusions in Figures S2 and S3. We tested whether the forced expression of *ste11* could recover sexual development in the CTDK-I deletion mutants. The overexpression of *ste11* from the *nmt1* promoter, which is roughly four to five times as strong as the physiological expression, effectively recovered conjugation and subsequent meiosis in *lsg1Δ*, *lsk1Δ* and *lsc1Δ* homothallic haploid cells (Figure S2C), indicating that the loss of *ste11* expression is a major cause of the mating and sporulation deficiency in the CTDK-I mutants. We then determined the range of genes whose expression is regulated by CTDK-I, by comparing the gene expression profiles between *lsg1Δ* and wild-type cells starved of nitrogen for 2.5 hours. Genome-wide microarray analysis indicated that the expression of 64 genes was downregulated more than two-fold in the *lsg1Δ* mutant, whereas 22 genes showed upregulation by more than two-fold in the mutant (Figure S3A). Notably, 33 out of the 64 downregulated genes identified, including *ste11* itself, have been shown previously to be controlled by Ste11 [19]. These genes are listed in Table S1. In contrast, the expression of *atf1*, *pcr1*, *rst2*, and other genes that also encode an upstream regulator of *ste11* transcription [20]–[25], was not significantly affected by the deletion of *lsg1* (Figure S3B), suggesting that CTDK-I may exert its effects on *ste11* transcription directly.

### Ser-2 of the Pol II CTD is phosphorylated by CTDK-I in the course of meiosis

**Figure 2:**
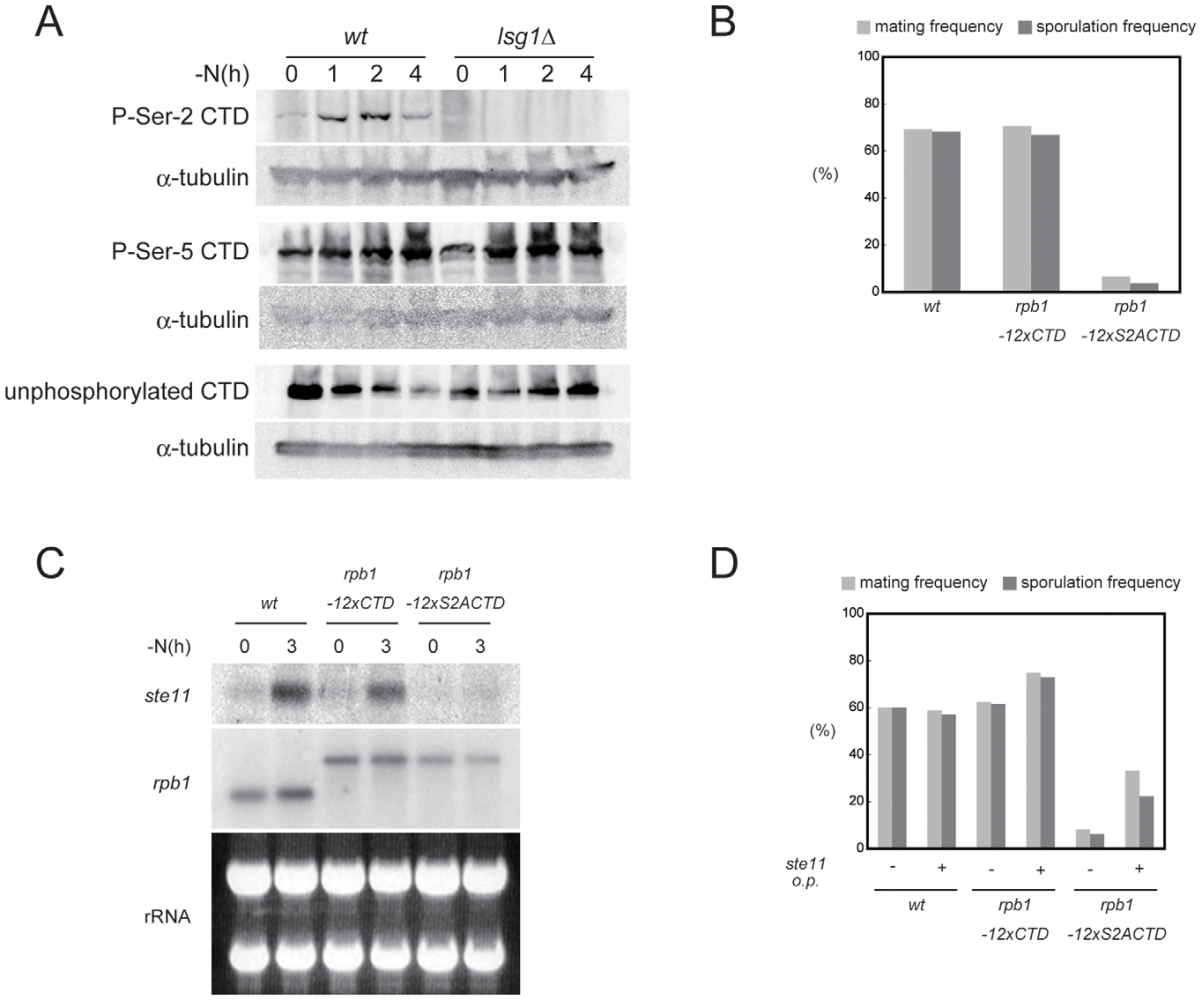
The phosphorylation of Ser-2 residues on Pol II CTD is required for *ste11* expression. (A) Nitrogen starvation induces the phosphorylation of Ser-2 residues on Pol II CTD in wild-type (JY450) but not in *lsg1Δ* (JT659) cells. Cells of the two strains were subjected to nitrogen starvation for the indicated periods and analyzed by immunoblotting with antibodies against Ser-2 phosphorylated CTD, Ser-5 phosphorylated CTD, or unphosphorylated CTD. α-tubulin is shown as a loading control. (B) Comparison of the mating and sporulation frequencies among wild-type (JY450), *rpb1-12×CTD* (JT668), and *rpb1-12×S2ACTD* (JT669) strains. Cells were incubated on SSA plates at 30°C for three days, and the frequencies were determined microscopically. (C) Expression of *ste11* in cells examined in (B). Cells were grown to mid-log phase and shifted to nitrogen-free medium. They were then harvested right before and at 4 hours after this shift, and subjected to northern blot analysis. rRNAs stained with ethidium bromide are shown as a loading control. Expression of *rbp1*, which was not affected by nitrogen starvation, is also shown for comparison. The *rbp1* transcripts in JT668 and JT669 were larger than the authentic transcript due to a vector sequence inserted during the strain construction [15]. (D) Effects of *ste11* overexpression on mating and sporulation in the *rpb1-12×CTD* and *rpb1-12×S2ACTD* strains. JY450, JT668, and JT669 cells harboring either pREP41-*ste11* or pREP41 were examined for their mating and sporulation frequencies after incubation on SSA plates at 30°C for three days.

Previous work has shown that Lsk1 is involved in the phosphorylation of Ser-2 residues within the heptad repeats of the carboxy terminal domain (CTD) of RNA polymerase II [15]. To determine whether the Pol II CTD phosphorylation status might be changed by the induction of sexual development, we analyzed phosphorylation of Ser-2 and Ser-5 residues within the CTD before and after the shift to nitrogen-depleted medium. Extracts were prepared from wild-type and *lsg1Δ* homothallic haploid cells, either growing or shifted to nitrogen-free minimal medium, and the phosphorylation of CTD was examined using monoclonal antibodies that recognize either phospho-Ser-2, phospho-Ser-5, or unphosphorylated CTD. As shown in Figure 2A, the phosphorylation of Ser-2 residues on the CTD repeats was increased by nitrogen starvation in wild-type cells, but not in *lsg1Δ* cells. The level of phospho-Ser-5 was unaffected by nitrogen starvation in both strains. These results suggest that nitrogen starvation induces the phosphorylation of CTD Ser-2 residues by CTDK-I.

We next evaluated the possibility that the insufficient phosphorylation of CTD Ser-2 residues in the CTDK-I mutants underlies their sexual development deficiency. For this purpose we examined the phenotypes caused by two *rpb1* alleles (reported by J. Karagiannis and kindly provided to us), namely *rpb1-12×CTD* and *rpb1-12×S2ACTD*. The former allele produces Rpb1 carrying a CTD that consists of 12 copies of the authentic heptad repeat (YSPTSPS), whereas the latter produces Rpb1 with 12 copies of a mutant heptad repeat in which Ser-2 is substituted by alanine (YAPTSPS) [15]. Wild-type Rpb1 carries 29 repeats of the heptad [26], but the previous work has shown that 12 repeats are sufficient for cell viability [15]. Cells carrying the *rpb1-12×S2ACTD* allele were impaired severely in terms of conjugation and sporulation (Figure 2B), and the transcription of *ste11* was greatly reduced in them (Figure 2C). Furthermore, the sterility of the *rpb1-12×S2ACTD* strain was rescued, although not completely, by the overexpression of *ste11* (Figure 2D). These results strongly suggest that CTDK-I facilitates the transcription of *ste11* by phosphorylating Ser-2 residues on Pol II CTD. In general, the *rpb1-12×S2ACTD* strain showed severer phenotypes than the CTDK-I mutants with regard to sexual development, probably because CTD Ser2 could also be phosphorylated supplementarily by Cdk9 [11].

### The stress-responsive MAP kinase pathway is required for the phosphorylation of CTD Ser-2 residues

We wished to determine the mechanism by which nitrogen starvation caused the increased phosphorylation of CTD Ser-2 by CTDK-I. The concentration of CTDK-I subunits per cell was not found to be significantly altered upon nitrogen starvation (Figure S4A). We also measured the levels of Fcp1, a phosphatase that has been shown to preferentially remove phosphate groups from synthetic CTD peptides phosphorylated on Ser-2 [27], [28]. However, the levels of this protein were also not changed significantly upon nitrogen starvation (Figure S4B).

**Figure 3:**
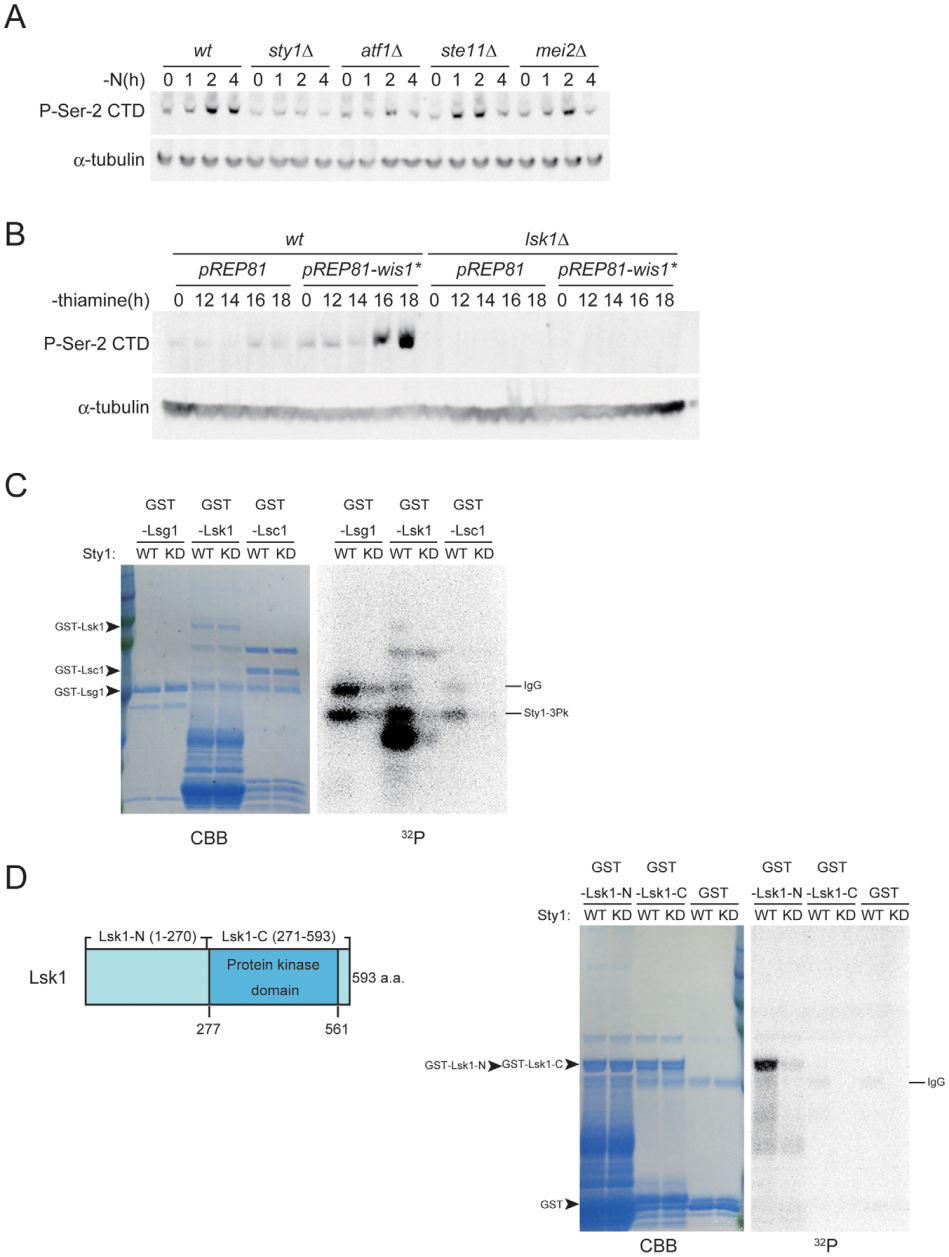
The stress-responsive MAP kinase Sty1 is essential for the phosphorylation of CTD Ser-2 residues. (A) CTD Ser-2 phosphorylation was examined in JY450 (wild-type), JT674 (*sty1Δ*), JX303 (*atf1Δ*), JZ496 (*ste11Δ*), and JZ127 (*mei2Δ*) cells. The cultures were subjected to nitrogen starvation, sampled at the indicated intervals, and analyzed by immunoblotting with antibodies specific for Ser-2 phosphorylated CTD. α-tubulin was detected as a loading control. (B) JY450 and JT660 (*lsk1Δ*) cells were transformed with either pREP81 or pREP81-wis1*, the latter of which expresses a constitutively active form of MAPKK Wis1. Each transformant was cultured in liquid MM without thiamine for 12 to 18 hours to derepress the weakened *nmt1* promoter on pREP81 (*nmt1-81*). Cells were harvested at the indicated times and analyzed by immunoblotting as in (A). (C) Phosphorylation of GST-Lsg1, GST-Lsk1 and GST-Lsc1 by Sty1 was examined *in vitro* (right panel, ^32^P). Substrates were stained with Coomassie brilliant blue to indicate their quantities (left panel, CBB). (D) GST-Lsk1-N (1–270), GST-Lsk1-C (271–593), and GST as a control were analyzed for phosphorylation (^32^P) and quantities (CBB) as in (C). A schematic illustration of the structure of Lsk1 is also shown.

It has been reported in *S. cerevisiae* that CTD Ser-2 phosphorylation increases both upon heat shock and during the diauxic shift [29]. The phosphorylation of CTD Ser-2 is also known to be elevated by an exposure to hydroxyurea or UV irradiation [30]. We speculated therefore that nitrogen starvation may be recognized as a stress, which could then affect the phosphorylation status of the CTD in fission yeast. We hence examined the possible involvement of Sty1 (also called Spc1/Phh1), a MAP kinase known to be crucial to the response to stress [31]–[33], in CTD phosphorylation. As shown in Figure 3A, the phosphorylation of CTD Ser-2 in response to nitrogen starvation was dramatically reduced in *sty1Δ* cells compared with wild-type cells. Deletion of the *atf1* gene, which encodes a target of Sty1 MAPK, also significantly affected Ser-2 phosphorylation, whereas the *ste11* and *mei2* genes appeared to be dispensable for this phosphorylation event in response to nitrogen starvation (Figure 3A). Deletion of *pcr1*, which encodes a bZIP protein that forms a heterodimer with Atf1 [21], [23], did not affect Ser-2 phosphorylation significantly (Figure S5), and produced a much less severe phenotype compared with mutants lacking *atf1*, as observed previously for other features [23], [34]. The deletion of *rst2*, which encodes a transcription factor necessary to activate *ste11* in response to glucose starvation and cAMP reduction [24], [25], also had no affect on Ser-2 phosphorylation (Figure S5).

We then examined the effects of a forced activation of the Sty1 MAPK pathway, by expressing a constitutively active form of Wis1 MAPKK in the yeast cells. Phosphorylation of Ser-2 was induced by expression of the active MAPKK from a plasmid, even in the presence of ample nitrogen (Figure 3B). However, this ectopic phosphorylation was not observed in *lsk1Δ* cells (Figure 3B), indicating that the observed phosphorylation was mediated by CTDK-I. These results suggest that the activation of Sty1 MAP kinase in response to nitrogen starvation is pivotal to the promotion of CTD Ser-2 phosphorylation by CTDK-I.

### Sty1 MAP kinase phosphorylates Lsk1, the catalytic subunit of CTDK-I *in vitro*

To examine if the stress-responsive MAPK Sty1 directly phosphorylates CTDK-I, we prepared an in vitro phosphorylation system as detailed in Materials and Methods. Each subunit of CTDK-I, namely Lsk1, Lsc1 or Lsg1, was fused with GST, and the fusion proteins were affinity-purified. Pk-tagged Sty1 MAPK (Sty1-Pk) and its kinase-dead form (Sty1-KD-Pk) were prepared respectively from *S. pombe* strains NJ761 and NJ767, provided kindly by N. Jones, as described previously [34]. The kinase preparation and each GST-fusion protein were mixed and incubated in the kinase reaction buffer supplemented with [γ-^32^P]-ATP. As shown in Figure 3C, GST-Lsk1 appeared to be phosphorylated by Sty1, although the full-length protein apparently underwent extensive proteolysis and a possible degradation product was the most heavily labeled. GST-Lsc1 and GST-Lsg1, as well as the control GST, did not appear to be a good substrate of Sty1 in this analysis (Figure 3C). To confirm that Sty1 could phosphorylate Lsk1, we divided Lsk1 into two parts, the N- and C-terminal halves, and fused each of them to GST (Figure 3D). These fusion proteins were relatively stable, and when mixed with active Sty1, the N-terminal half was significantly phosphorylated (Figure 3D). Moreover, our preliminary analysis has shown that at least serine 109 on Lsk1, which constitutes a MAPK substrate consensus sequence PGSP, is a preferred phosphorylation site for Sty1 (data not shown). Analysis of Lsg1 dissected into two parts confirmed that it was not likely to be a substrate of Sty1 (data not shown). These results indicate that Sty1 MAPK is likely to phosphorylate Lsk1 directly and thereby activate CTDK-I, which in turn phosphorylates CTD Ser-2 residues.

### The phosphorylation of CTD Ser-2 is regulated by a feedback system during meiosis

**Figure 4:**
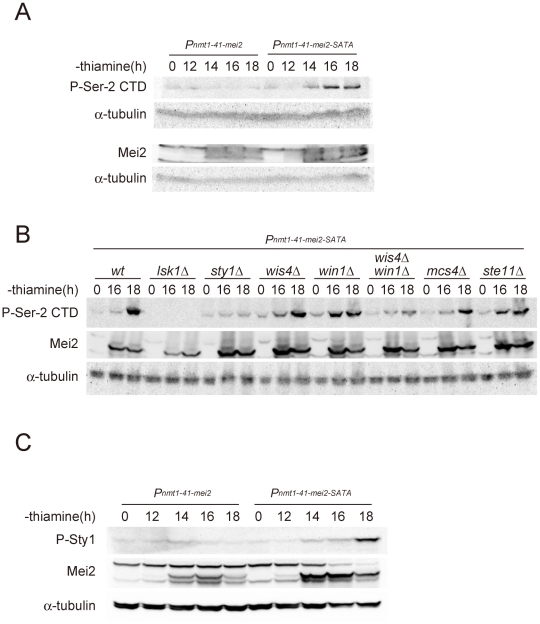
The activation of Mei2 leads to elevated CTD Ser-2 phosphorylation. (A) Cells of the JX382 fission yeast strain, which carries a *mei2* ORF driven by the attenuated *nmt1* promoter (*nmt1-41*) on the chromosome, and of the JX383 strain, which carries the *mei2-SATA* ORF but is otherwise identical to JX382, were cultured in liquid MM with no supplementation of thiamine. The *nmt1-41* promoter was therefore derepressed under these growth conditions. Cells were sampled at the indicated times and the phosphorylation of Ser-2 residues within the CTD repeats was examined by immunoblotting. These samples were also examined for the expression of Mei2 protein by western blot. α-tubulin is shown as a loading control. (B) The *Pnmt1-41-mei2-SATA* allele in JX383 was combined with either *lsk1Δ* (JT675), *sty1Δ* (JT676), *wis4Δ* (JT677), *win1Δ* (JT678), *wis4Δ win1Δ* (JT679), *mcs4Δ* (JT680), or *ste11Δ* (JT681). Cells of each strain were cultured in liquid MM with no thiamine addition for 16 and 18 hours, and harvested. Lysates were prepared and then analyzed by immunoblotting with anti-phospho-Ser-2 CTD. The production of Mei2 protein was also evaluated by immunoblotting with α-tubulin detected as a loading control. (C) Cells of the JX382 and JX383 strains were cultured and processed as described in (A). Immunoblotting was performed using antibodies specific for the phosphorylated form of Sty1 MAPK. The Mei2 protein and a loading control α-tubulin were also immunoblotted.

We made a surprising observation when we analyzed the status of CTD Ser-2 phosphorylation in cells undergoing ectopic meiosis induced by artificial expression of the activated form of Mei2, i.e., Mei2-SATA. As we reported previously [6], these cells underwent meiosis in the presence of ample nitrogen, a condition that does not stimulate the stress-responsive Sty1 MAP kinase cascade. However, the phosphorylation of CTD Ser-2 was observed in these meiotic cells (Figure 4A). Given this finding, we speculated as to whether the phosphorylation of CTD Ser-2 during Mei2-SATA-induced meiosis was dependent on CTDK-I and/or Sty1. We further tested relevant mutant strains and found that the Mei2-SATA-induced Ser-2 phosphorylation was abolished in *lsk1Δ* and reduced dramatically in *sty1Δ*, indicating its stringent dependency on both of these factors (Figure 4B). Sty1 has been positioned upstream of *mei2* expression in the stress-responsive signal transduction pathway and in cooperation with a chromatin-remodeling factor Atf1, activates the transcription of *ste11* [20]–[22]. The produced Ste11 in turn binds to the upstream region of *mei2* and activates the transcription of this gene [16]. We thus hypothesized that activated Mei2 can affect its upstream factors through a feedback regulation.

To identify the component of the stress-responsive signaling pathway that is feedback-regulated by Mei2, we examined mutants that are defective in components of the pathway that function upstream of Sty1. Sty1 MAPK is activated by Wis1 MAPKK [31], [32], [35], [36], which in turn is activated by either Wis4/Wak1 MAPKKK or Win1 MAPKK [37]–[39]. A response regulator protein, Mcs4, associates with Wis4/Wak1, and probably also with Win1, to regulate the MAPKKK activity [38], [40]. We investigated the phosphorylation of Ser-2 during Mei2-SATA-induced meiosis in *mcs4Δ*, *wis4Δ*, *win1Δ*, and *wis4Δ win1Δ* mutant strains, together with control wild-type, *lsk1Δ*, *sty1Δ*, and *ste11Δ* strains. As summarized in Figure 4B, the phosphorylation of Ser-2 was observed in *mcs4Δ* and *ste11Δ* cells, indicating that Mcs4 and Ste11 are not directly involved in the feedback activation of Ser-2 phosphorylation. Ser-2 phosphorylation was observed also in the *wis4Δ* and *win1Δ* mutants but was found to be greatly reduced in the *wis4Δ win1Δ* double mutant. These results indicated that the feedback signals from activated Mei2 might ultimately merge with the stress-responsive MAPK cascade at the Wis4/Wak1 and Win1 MAPKKKs, although there could be a third target because Ser-2 phosphorylation was not completely abolished in *wis4Δ win1Δ* (Figure 4B). We observed that the level of Sty1 MAPK phosphorylation increased during Mei2-SATA-induced meiosis (Figure 4C), which reinforces the presence of a signaling pathway from Mei2 to the MAPK cascade.

### Physiological significance of the feedback

**Figure 5:**
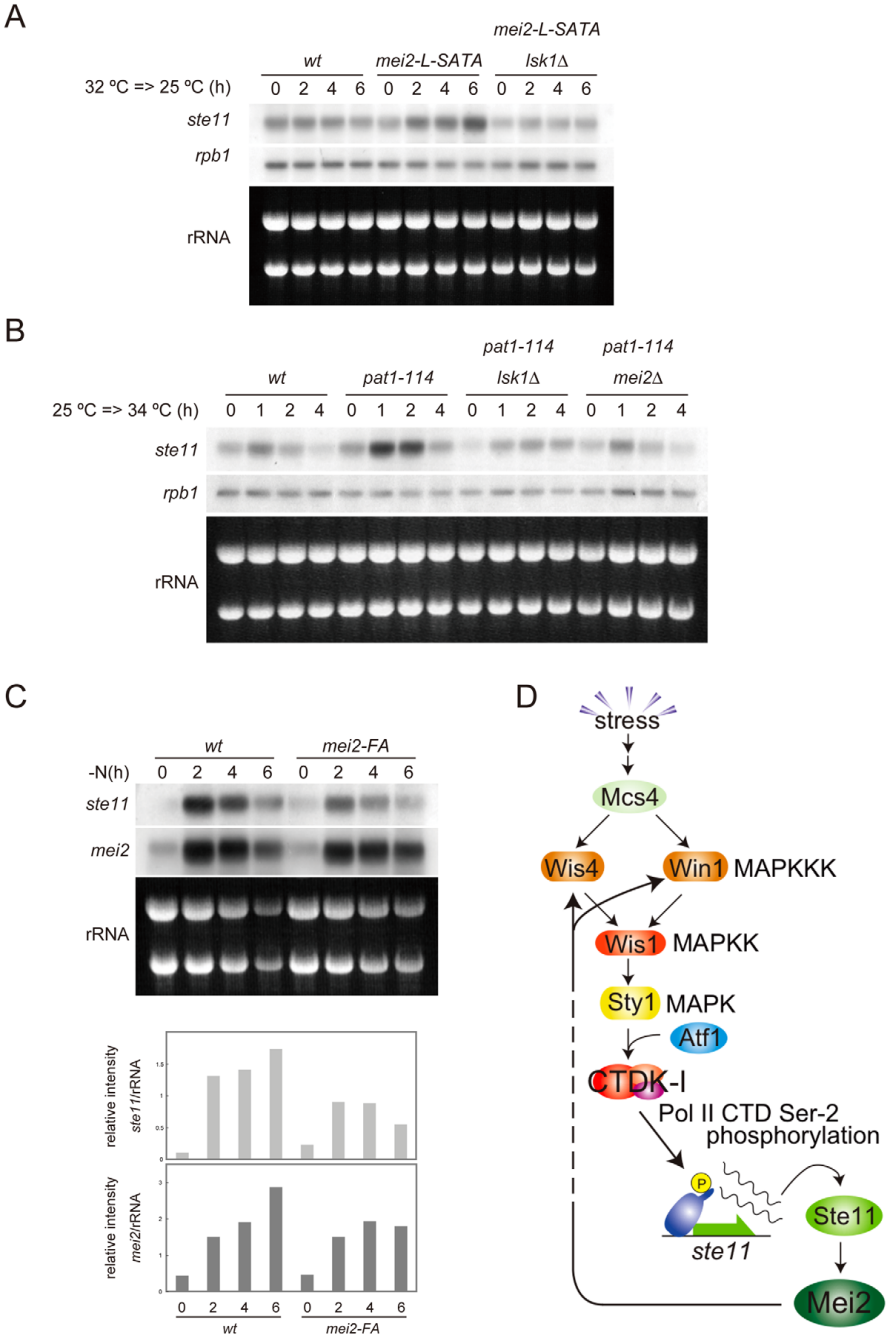
Activated Mei2 enhances expression of *ste11* via CTDK-I. (A) Northern blot analysis of *ste11* expression in JY741 (wild-type), JV312 (*mei2-L-SATA*) and JT663 (*mei2-L-SATA lsk1Δ*). Cells were grown to the mid-log phase in MM at 32°C, shifted to 25°C, and sampled at the indicated intervals. Total RNA (10 µg) from each sample was resolved by gel electrophoresis and subjected to northern blot analysis to detect transcripts of *ste11* and *rpb1*. rRNAs stained with ethidium bromide are shown as a loading control. (B) Northern blot analysis of *ste11* expression in JY333 (wild-type), JZ409 (*pat1-114*), JT915 (*pat1-114 lsk1Δ*) and JW92 (*pat1-114 mei2Δ*). Cells were grown to the mid-log phase in MM at 25°C, shifted to 34°C, sampled at the indicated intervals, and analyzed as in (A). (C) Northern blot analysis of *ste11* and *mei2* expression in heterozygous diploid (*h* ^+^/*h* ^−^) strains JY362 (wild-type) and JT908 (*mei2-FA/mei2-FA*). Cells were grown to the mid-log phase at 30°C, shifted to nitrogen-free medium, and sampled at the indicated intervals. Total RNA (10 µg) from each sample was resolved by gel electrophoresis and subjected to northern blot analysis to detect *ste11* and *mei2* transcripts. The level of their expression was normalized by the amount of rRNAs stained with ethidium bromide, and is displayed in a graph for quantitative comparison (lower panel). (D) A diagram of the regulatory pathway leading to the activation of CTDK-I and expression of *ste11*, which incorporates a feedback loop from Mei2 to MAPKKK Wis4 and Win1.

To evaluate physiological significance of the feedback, we examined whether activation of Mei2 would result in enhancement of *ste11* expression during meiosis. Firstly, we induced ectopic meiosis by shifting the mei2-L-SATA strain from 32°C to 25°C in the presence of rich nutrition. As shown in Figure 5A, expression of *ste11* was evident in this strain but not in the wild-type, and this expression was dependent on *lsk1*. Secondly, we induced ectopic meiosis by shifting the temperature-sensitive *pat1-114* mutant from 25°C to 34°C. Again, expression of *ste11* was induced significantly in *pat1-114* cells under rich nutrition, in an *lsk1*-dependent manner (Figure 5B). Deletion of *mei2* blocked *ste11* expression in these cells. The temperature-shift did not induce *ste11* expression in wild-type (Figure 5B) or *mei2Δ* cells (not shown). These results indicate clearly that activation of Mei2 can stimulate expression of *ste11* through phosphorylation of PolII CTD.

We finally evaluated the contribution of the feedback regulation to the expression of *ste11* during meiosis under physiological conditions. To do so, we used the *mei2-FA* allele, which produces inactive Mei2 protein [5], [6]. We compared expression of *ste11* and *mei2* in wild-type and *mei2-FA* cells subjected to nitrogen starvation. As shown in Figure 5C, the level of *ste11* mRNA, normalized by ribosomal RNA, and that of *mei2* mRNA also, were higher in wild-type cells than in *mei2-FA* cells, and the difference became greater in later stages. This suggests that activated Mei2 protein in wild-type cells indeed enhances *ste11* expression via feedback.

Taken together, we propose that fission yeast possess a regulatory circuit, as depicted in Figure 5D, which is likely to be crucial in ensuring an irreversible commitment to meiosis and a strict differentiation of the mitotic and meiotic cell cycle programs.

## Discussion

In our present study, we have demonstrated that a genetic interaction exists between the subunits of CTDK-I, a protein kinase complex that phosphorylates RNA polymerase II CTD, and the master meiotic regulator in fission yeast, Mei2. Furthermore, our analyses indicate that a loss of CTDK-I function impairs the transcription of the *ste11* gene, which encodes a transcription activator essential for the expression of *mei2* and other genes crucial for sexual development. However, this loss of function does not significantly affect the gene expression required for vegetative growth. In an independent study, Hermand and colleagues have performed genome-wide mapping of three kinds of CTD kinases and also of serine 2- and 5-phosphorylated Pol II in fission yeast to investigate the link between CTD phosphorylation and specific cellular events [11]. Consequently they have found that the CTDK-I catalytic subunit Lsk1 and Ser-2-phosphorylated Pol II associate with a rather limited number of transcription units and play only minor roles during vegetative growth, but become essential during sexual development. These authors have further reported that nitrogen starvation enhances recruitment of Lsk1 to the *ste11* gene, and remarked that the phosphorylation of CTD Ser-2 plays a highly specialized role in gene regulation in fission yeast, unlike in other organisms, and is virtually confined to the regulation of a single key gene controlling sexual differentiation. Our study fully supports this notion. While a subsequent study [26] suggests that the deleterious effects of loss of Ser-2 phosphorylation on *ste11* transcrition can be compensated partially by loss of Ser-7 phosphorylation, the nature of such extreme specification and its evolution is an intriguing enigma.

Our present data have further shown that the stress-responsive MAP kinase pathway is crucial for the activation of CTDK-I under conditions of nitrogen starvation. The requirement for Sty1 MAPK and its target Atf1 for the expression of *ste11* has been known for some time [20]–[22], but the details of the molecular mechanisms involved have remained unknown. It now appears that CTD Ser-2 phosphorylation is a key step in the activation of *ste11* expression by the Sty1 MAPK cascade. It has been shown that when phosphorylated and activated by Wis1 MAPKK, the Sty1 protein migrates to the nucleus and resides on the promoter regions of stress-responsive genes [31], [34], [41]. This is also the case for the Sty1 ortholog in *S. cerevisiae* Hog1 [42], [43]. As shown above, Sty1 can directly phosphorylate Lsk1 *in vitro*. While the phosphorylation of Lsk1 *in vivo* remains to be confirmed, it appears to be conceivable that Sty1 may also be recruited to the *ste11* promoter and phosphorylate CTDK-I staying there, which in turn phosphorylates CTD and licenses RNA polymerase II to transcribe the gene. In this regard, it is noteworthy that *hsp9*, which encodes a small heat-shock protein [44] and is one of the genes responsible for the “core environmental stress response” or CESR in fission yeast [45], was detected among our possible target genes upregulated by CTD Ser-2 phosphorylation (Table S1). Interestingly, Reiter et al. have shown previously that Sty1 MAPK is recruited to the promoter of *hsp9* and other CSRE genes upon osmotic stress in an Atf1-dependent manner, but does not necessarily phosphorylate Atf1 as a substrate [34]. This suggests that *ste11* and *hsp9* may be similarly regulated by the Sty1 – CTDK-I – CTD phosphorylation system. However, conventional Chip analyses have not provided convincing evidence for the association of Sty1 with the *ste11* promoter, and we are conducting further experiments to scrutinize this possible scheme.

The results of our present analyses demonstrate unambiguously that a feedback-regulatory system operates in fission yeast during the meiotic cell cycle. In this feedback loop, the active form of Mei2 can eventually stimulate the stress-responsive MAPKKKs and enhance the transcription of *ste11* through the Sty1 – CTDK-I – CTD phosphorylation system. From our findings we can outline a framework of the molecular mechanisms that differentiate the mitotic and meiotic programs in fission yeast as in Figure 5D. However, it remains currently unknown how the RNA-binding protein Mei2 can fulfill such a never-anticipated task and how many steps may mediate between Mei2 and the MAPKKKs, raising another challenging scientific query as represented by the broken line in Figure 5D.

## Materials and Methods

### Fission yeast strains, genetic procedures, and media

The *S. pombe* strains used in this study are listed in Table S2. The general genetic procedures used in the *S. pombe* experiments were as described previously [46]. Complete medium YE, minimal medium SD, minimal medium MM and its nitrogen-free derivative MM-N [47], synthetic sporulation medium SSA [48] were used to culture the cells. Transformation of *S. pombe* was performed using the lithium acetate method [49].

### Genetic screen

The *ura4* ^+^ cassette used for insertion mutagenesis was amplified by PCR using the primers N_18_ AGCTTAGCTACAAATCCCACTGGCT and N_18_ TGTGATATTGACGAACTTTTTGAC (N_18_: 18b random DNA sequence). The PCR products were then introduced into JV312 (*mei2-L-SATA ura4-D18*) cells, and transformants were plated onto SD lacking uracil and incubated at 25°C. Colonies were selected, and the site of *ura4* ^+^ integration was determined via the sequencing of inverse PCR products [50].

### Flow cytometric analysis

Samples were prepared for flow cytometry essentially as described previously [51] and then analyzed using a FACScan (Becton-Dickinson, San Jose, CA).

### Microarray analysis

JY450 (wild-type) and JT659 (*lsg1Δ*) cells were grown to mid-log phase in MM medium and shifted to MM-N medium. The cells were collected 2.5 h after the shift, and total RNA was extracted as described previously [52]. Data acquisition and normalization were performed by Roche Applied Science, Japan. The microarray data was deposited to the GEO database under the accession number of GSE32516.

### Northern blot analysis

Northern blot analysis was performed as described [53]. DNA fragments used to probe for transcripts of *ste11*, *rpb1* and *mei2* were labeled with [α-32P] dCTP using random primers.

### Western blot analysis

Cell extracts were prepared and separated essentially as described earlier [54]. Briefly, cells grown to the mid-log phase were shifted to nitrogen-free medium, and sampled at various intervals. Total lysates were extracted and resolved by SDS-PAGE. Immunoblotting was performed using primary antibodies specific to unphosphorylated CTD (8WG16, Covance, Princeton, NJ, used at 1∶2000), Ser-5 phosphorylated CTD (H14, Covance, used at 1∶2000), Ser-2 phosphorylated CTD (H5, Covance, used at 1∶1000), Mei2 (Our lab preparation, used at 1∶1000), the phosphorylated form of Sty1 MAPK (P-p38 MAPK, Cell Signaling Technology, Danvers, MA, used at 1∶500), or GFP (clones 7.1 and 13.1, Roche Applied Science, Indianapolis, IN, used at 1∶1000). As secondary antibodies, donkey anti-rabbit IgG conjugated with horseradish peroxidase (GE Healthcare, Waukesha, WI) was used for the Mei2 and P-p38 MAPK antibodies at a dilution of 1∶2000. Sheep anti-mouse IgG conjugated with horseradish peroxidase (GE Healthcare) was used to detect all other primary antibodies at a dilution of 1∶2000. Immunoblotting with a monoclonal antibodies specific for α-tubulin, either TAT-1 (a gift from Dr. Keith Gull, University of Birmingham) [55], or Clone B-5-1-2 (Sigma Aldrich, St. Louis, MO), was performed as a loading control.

### *In vitro* phosphorylation assay

Cells expressing chromosomally tagged Sty1-3Pk (NJ761), or Sty1KD-3Pk (NJ767) were subjected to nitrogen starvation for 1 h. Extracts were prepared, protein immunoprecipitated, and the immuno-complexes tested for kinase activity as described [34]. Affinity purified GST-fusion proteins were used as substrates.

## Supporting Information

Figure S1 Comparison of *S. pombe* Lsg1 with its *S. cerevisiae* orthologue CTK3. A ClustalW alignment of Lsg1 and CTK3 is shown.
(TIF)

Figure S2 Deficiency of the *lsg1Δ* mutant in sexual development. (A) FACS analysis of DNA content in cells subjected to nitrogen starvation. Cells of diploid strains JY362 (wild-type), JT665 (*lsg1Δ*), JZ403 (*ste11Δ*) and JY776 (*mei2Δ*) cultured in liquid MM medium to mid-log phase were shifted to MM-N medium. Aliquots were taken at indicated time and the DNA content per cell was determined by FACS analysis. (B) Northern blot analysis of *ste11* and *rbp1* in JY450 (wild-type) and JT659 (*lsg1Δ*) cells. The cultures were grown to the mid-log phase, shifted to nitrogen-free medium, and sampled at the indicated intervals. Total RNA (10 µg) from each sample was resolved by gel electrophoresis and subjected to northern blot analysis to detect *ste11* and *rbp1* transcripts. rRNAs stained with ethidium bromide are shown as a loading control. (C) Recovery of mating and subsequent sporulation by *ste11* overexpression in the CTDK-I deletion mutants. Cells of the homothallic haploid strains JY450 (wild-type), JZ396 (*ste11Δ*), JT659 (*lsg1Δ*), JT660 (*lsk1Δ*), and JT661 (*lsc1Δ*), harboring either the pREP41-*ste11* or control pREP41 vectors, were examined for mating and subsequent sporulation after incubation on SSA plates at 30°C for three days.
(TIF)

Figure S3 Expression of *ste11* is a major target of CTDK-I. (A) Comparison of the global gene expression profiles between the wild-type (JY450) and *lsg1Δ* (JT659) fission yeast strains. Cells of each strain were grown to the mid-log phase in liquid MM and shifted to nitrogen-free MM-N. The cells were harvested 2.5 h after this shift. RNA was prepared from each sample and analyzed on a DNA microarray covering 4,997 genes. (B) Relative changes in gene expression levels caused by loss of *lsg1* function. The *ste11* gene and genes encoding its upstream regulators on the cAMP and stress-responsive MAPK pathways were examined. The values are presented using binary logarithms.
(TIF)

Figure S4 Quantification of subunits of CTDK-I and a phosphatase Fcp1 in cells subjected to nitrogen starvation. (A) Strains expressing either *lsg1-gfp* (JT670), *lsk1-gfp* (JT671), or *lsc1-gfp* (JT672) were grown to the mid-log phase and shifted to nitrogen-free medium. Cells were sampled at indicated time, and subjected to immunoblotting with antibody specific for GFP. α-tubulin is shown as a loading control. (B) A strain expressing *fcp1-gfp* (JT673) was analyzed as in (A).
(TIF)

Figure S5 Pcr1 and Rst2 do not contribute to CTD Ser-2 phosphorylation significantly. Wild-type (JY450), *pcr1Δ* (JX25), and *rst2Δ* (JX231) cells subjected to nitrogen starvation were examined for CTD Ser-2 phosphorylation by immunoblotting with antibody specific for the Ser-2 phosphorylated form of the CTD (H5). α-tubulin is shown as a loading control.
(TIF)

Table S1 Genes downregulated in the *lsg1*-deletion mutant. Genes regulated by Ste11 are marked in blue, and *hsp9* is marked in yellow.
(XLS)

Table S2 *S. pombe* strains used in this study.
(DOC)
