# Supplementary figures and images for: The Fission Yeast Stress-Responsive MAPK Pathway Promotes Meiosis via the Phosphorylation of Pol II CTD in Response to Environmental and Feedback Cues
Source: PLoS Genet. 2011 Dec 1;7(12):e1002387. doi: 10.1371/journal.pgen.1002387 (PMC3228818; doi:10.1371/journal.pgen.1002387)

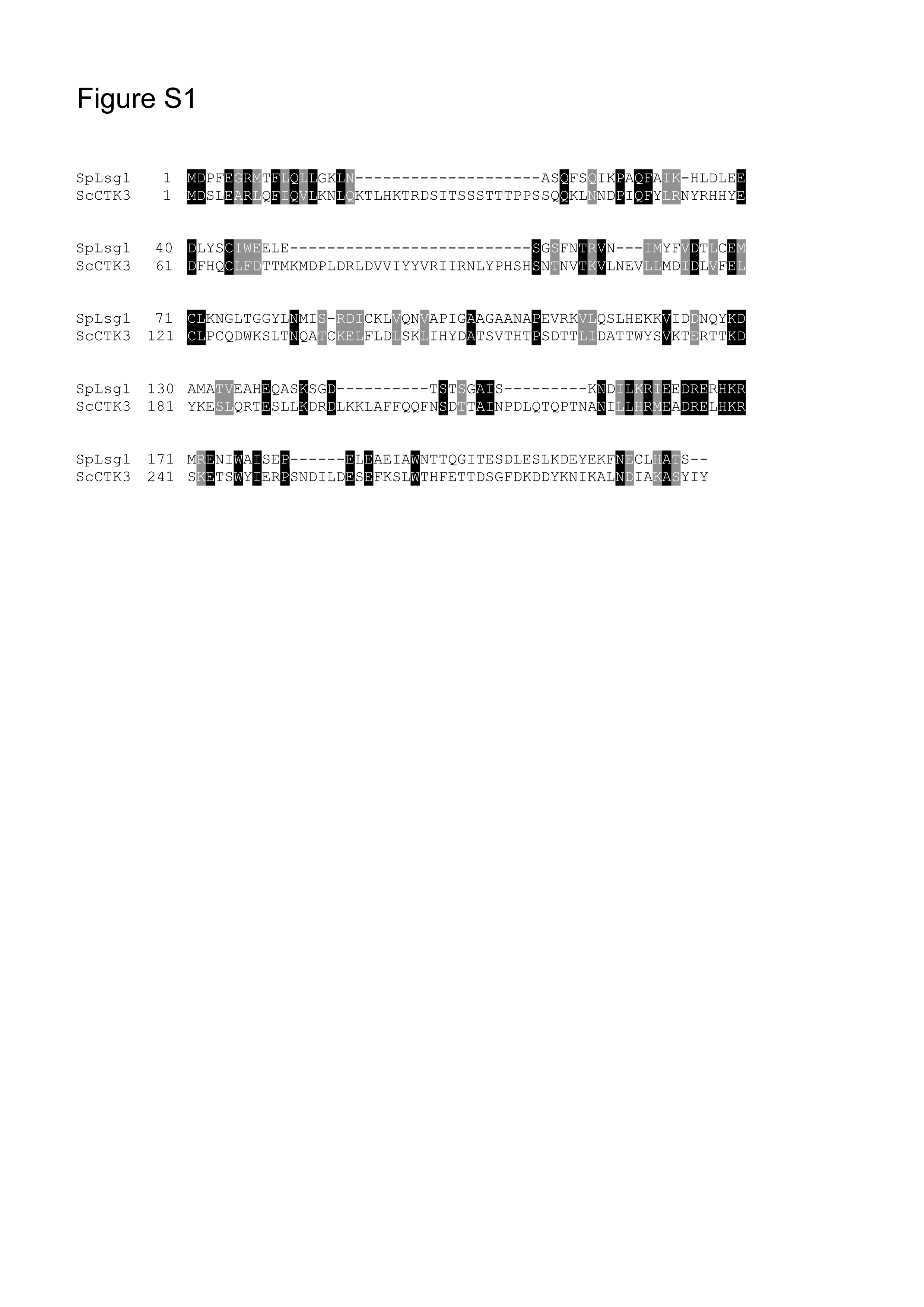

Supplement: Figure S1 — Comparison of S. pombe Lsg1 with its S. cerevisiae orthologue CTK3. A ClustalW alignment of Lsg1 and CTK3 is shown. (TIF) [file pgen.1002387.s001.tif]

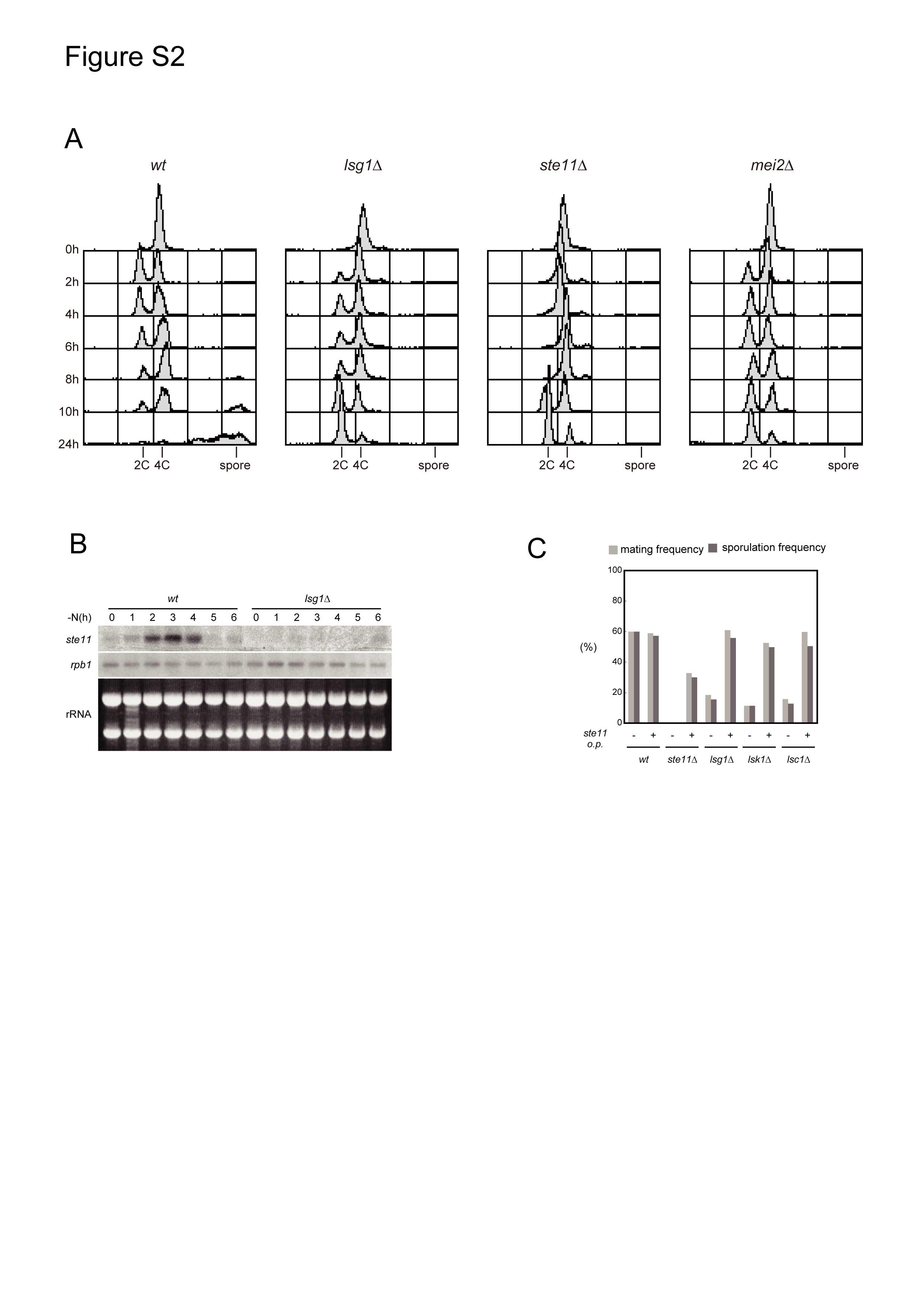

Supplement: Figure S2 — Deficiency of the lsg1Δ mutant in sexual development. (A) FACS analysis of DNA content in cells subjected to nitrogen starvation. Cells of diploid strains JY362 (wild-type), JT665 (lsg1Δ), JZ403 (ste11Δ) and JY776 (mei2Δ) cultured in liquid MM medium to mid-log phase were shifted to MM-N medium. Aliquots were taken at indicated time and the DNA content per cell was determined by FACS analysis. (B) Northern blot analysis of ste11 and rbp1 in JY450 (wild-type) and JT659 (lsg1Δ) cells. The cultures were grown to the mid-log phase, shifted to nitrogen-free medium, and sampled at the indicated intervals. Total RNA (10 µg) from each sample was resolved by gel electrophoresis and subjected to northern blot analysis to detect ste11 and rbp1 transcripts. rRNAs stained with ethidium bromide are shown as a loading control. (C) Recovery of mating and subsequent sporulation by ste11 overexpression in the CTDK-I deletion mutants. Cells of the homothallic haploid strains JY450 (wild-type), JZ396 (ste11Δ), JT659 (lsg1Δ), JT660 (lsk1Δ), and JT661 (lsc1Δ), harboring either the pREP41-ste11 or control pREP41 vectors, were examined for mating and subsequent sporulation after incubation on SSA plates at 30°C for three days. (TIF) [file pgen.1002387.s002.tif]

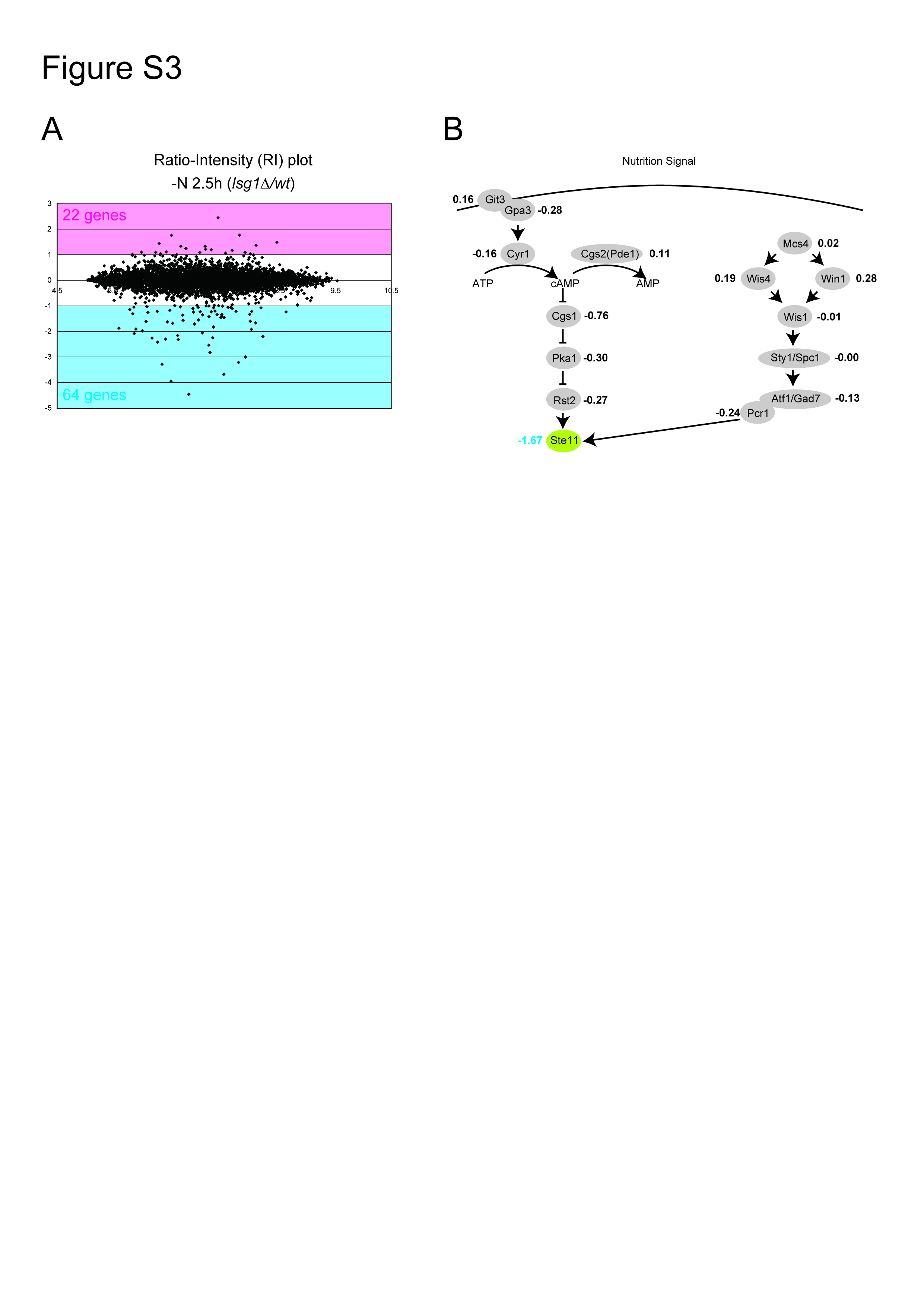

Supplement: Figure S3 — Expression of ste11 is a major target of CTDK-I. (A) Comparison of the global gene expression profiles between the wild-type (JY450) and lsg1Δ (JT659) fission yeast strains. Cells of each strain were grown to the mid-log phase in liquid MM and shifted to nitrogen-free MM-N. The cells were harvested 2.5 h after this shift. RNA was prepared from each sample and analyzed on a DNA microarray covering 4,997 genes. (B) Relative changes in gene expression levels caused by loss of lsg1 function. The ste11 gene and genes encoding its upstream regulators on the cAMP and stress-responsive MAPK pathways were examined. The values are presented using binary logarithms. (TIF) [file pgen.1002387.s003.tif]

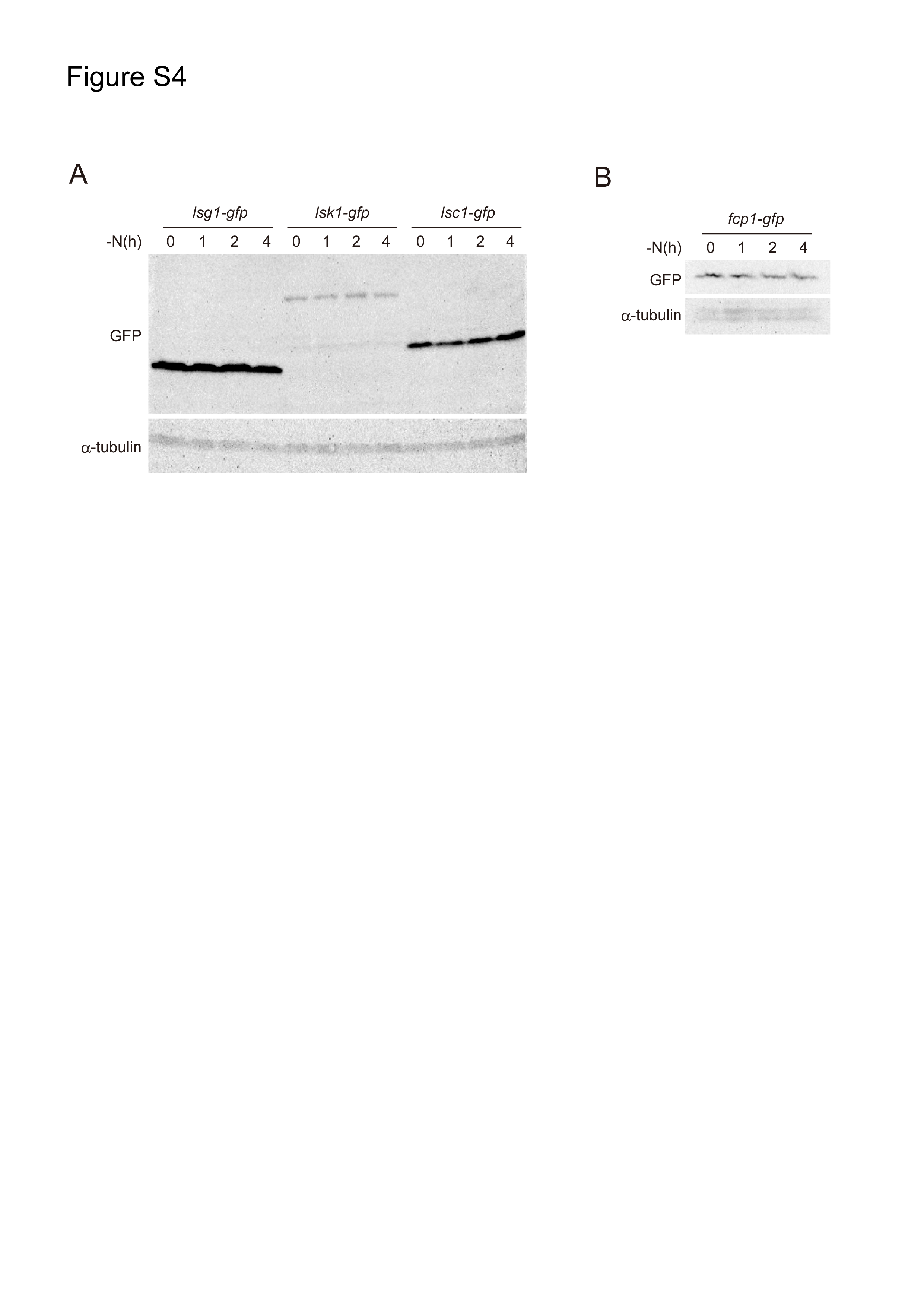

Supplement: Figure S4 — Quantification of subunits of CTDK-I and a phosphatase Fcp1 in cells subjected to nitrogen starvation. (A) Strains expressing either lsg1-gfp (JT670), lsk1-gfp (JT671), or lsc1-gfp (JT672) were grown to the mid-log phase and shifted to nitrogen-free medium. Cells were sampled at indicated time, and subjected to immunoblotting with antibody specific for GFP. α-tubulin is shown as a loading control. (B) A strain expressing fcp1-gfp (JT673) was analyzed as in (A). (TIF) [file pgen.1002387.s004.tif]

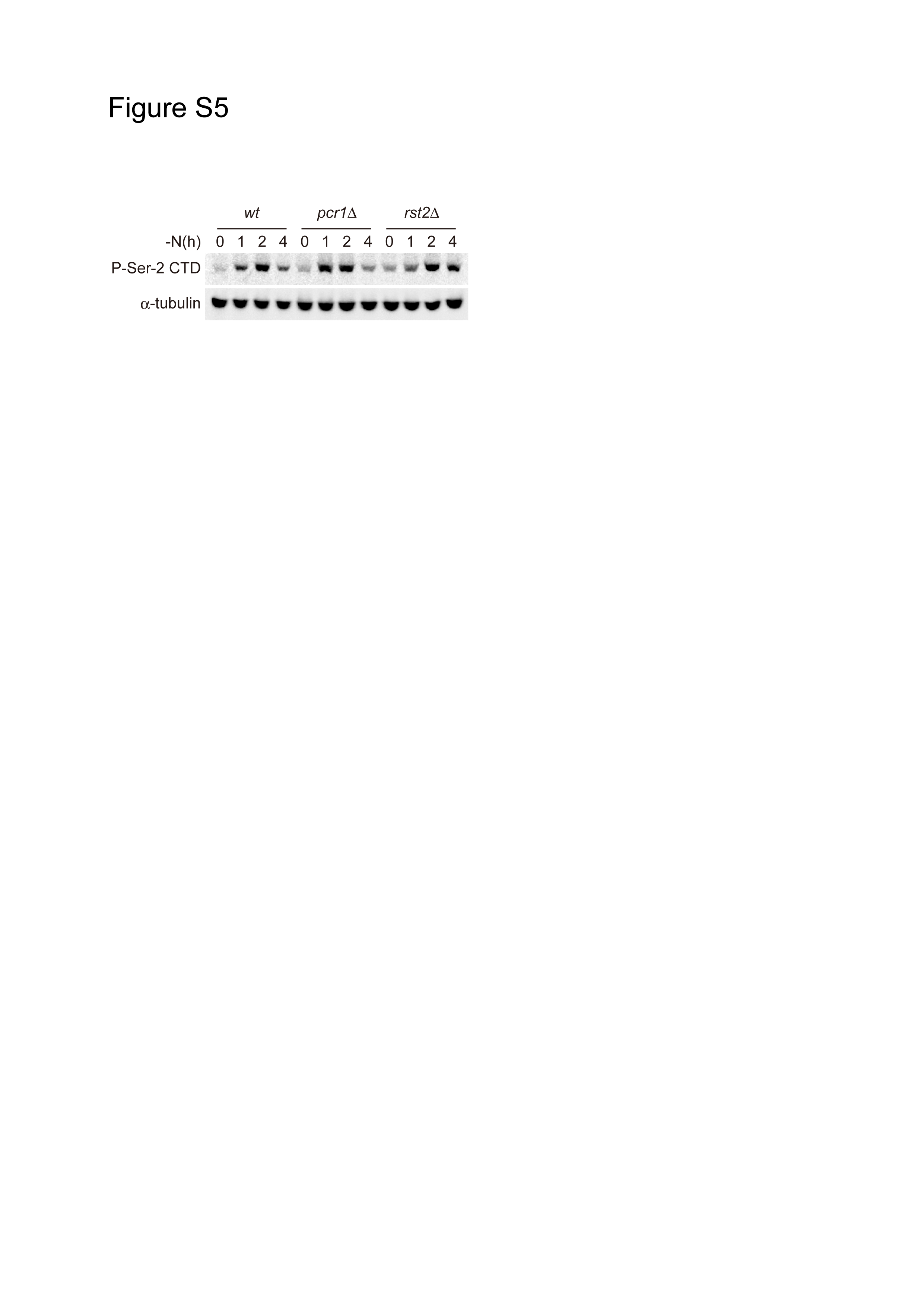

Supplement: Figure S5 — Pcr1 and Rst2 do not contribute to CTD Ser-2 phosphorylation significantly. Wild-type (JY450), pcr1Δ (JX25), and rst2Δ (JX231) cells subjected to nitrogen starvation were examined for CTD Ser-2 phosphorylation by immunoblotting with antibody specific for the Ser-2 phosphorylated form of the CTD (H5). α-tubulin is shown as a loading control. (TIF) [file pgen.1002387.s005.tif]
