# Supplementary material for: The Fission Yeast Stress-Responsive MAPK Pathway Promotes Meiosis via the Phosphorylation of Pol II CTD in Response to Environmental and Feedback Cues
Source: PLoS Genet. 2011 Dec 1;7(12):e1002387. doi: 10.1371/journal.pgen.1002387 (PMC3228818; doi:10.1371/journal.pgen.1002387)
Supplement: Table S2 — S. pombe strains used in this study. (DOC) [file pgen.1002387.s007.doc]

**Table S2. *S. pombe*** strains used in this study

| Strain | Genotype |
| --- | --- |

JT659 *h^90^ lsg1::kan ade6-M216 leu1*

JT660 *h^90^ lsk1::kan ade6-M216 leu1*

JT661 *h^90^ lsc1::kan ade6-M216 leu1*

JT662 *h^-^ lsg1::kan mei2::mei2-L-SATA ade6-M216 leu1 ura4-D18*

JT663 *h^-^ lsk1::kan mei2::mei2-L-SATA ade6-M216 leu1 ura4-D18*

JT664 *h^-^ lsc1::kan mei2::mei2-L-SATA ade6-M216 leu1 ura4-D18*

JT665 *h^+^/h^-^ lsg1::kan/lsg1::kan ade6-M210/ade6-M216 leu1/leu1*

JT666 *h^+^/h^-^* *lsk1::kan/lsk1::kan ade6-M210/ade6-M216 leu1/leu1*

JT667 *h^+^/h^-^ lsc1::kan/lsc1::kan ade6-M210/ade6-M216 leu1/leu1*

JT668 *h^90^ rpb1-12xCTD<<ura4^+^ ade6-M216 leu1 ura4-D18*

JT669 *h^90^ rpb1-12xS2ACTD<<ura4^+^ ade6-M216 leu1 ura4-D18*

JT670 *h^90^ lsg1-GFP<<kan ade6-M216 leu1*

JT671 *h^90^ lsk1-GFP<<kan ade6-M216 leu1*

JT672 *h^90^ lsc1-GFP<<kan ade6-M216 leu1*

JT673 *h^90^ fcp1-GFP<<kan ade6-M216 leu1*

JT674 *h^90^ sty1::kan ade6-M216 leu1*

JT675 *h^-^ lsk1::kan leu1::P41nmt1-mei2-SATA<<ura4^+^ ura4-D18 ade6-M210*

JT676 *h^-^ sty1::kan leu1::P41nmt1-mei2-SATA<<ura4^+^ ura4-D18 ade6-M210*

JT677 *h^-^ wis4::kan leu1::P41nmt1-mei2-SATA<<ura4^+^ ura4-D18 ade6-M210*

JT678 *h^-^ win1::hph leu1::P41nmt1-mei2-SATA<<ura4^+^ ura4-D18 ade6-M210*

JT679 *h^-^ wis4::kan win1::hph leu1::P41nmt1-mei2-SATA<<ura4^+^ ura4-D18 ade6-M210*

JT680 *h^-^ mcs4::kan leu1:: P41nmt1-mei2-SATA<<ura4^+^ ura4-D18 ade6-M210*

JT681 *h^-^ ste11::kan leu1:: P41nmt1-mei2-SATA<<ura4^+^ ura4-D18 ade6-M210*

JT908 *h^+^/h^-^ mei2-FA/mei2-FA ade6-M210/ade6-M216 leu1/leu1*

JT915 *h^-^ lsk1::kan pat1-114 ade6-M216 leu1*

JV312 *h^-^ mei2::mei2-L-SATA ade6-M216 leu1 ura4-D18*

JW92 *h^-^ mei2::ura4^+^ pat1-114 ade6-M216 leu1 ura4-D18*

JX25 *h^90^ pcr1::ura4^+^ ade6-M216 leu1 ura4-D18*

JX231 *h^90^ rst2::ura4^+^ ade6-M216 leu1 ura4-D18*

JX303 *h^90^ atf1::ura4^+^ ade6-M216 leu1 ura4-D18*

JX382 *h^-^ leu1:: P41nmt1-mei2<<ura4^+^ ura4-D18 ade6-M210*

JX383 *h^-^ leu1::P41nmt1-mei2-SATA<<ura4^+^ ura4-D18 ade6-M210*

JY333 *h^-^ ade6-M216 leu1*

JY362 *h^+^/h^-^ ade6-M210/ade6-M216 leu1/leu1*

JY741 *h^-^ ade6-M216 leu1 ura4-D18*

JY775 *h^-^ mei2::ura4^+^ ade6-M216 leu1 ura4-D18*

JY776 *h^+^/h^-^ mei2::ura4^+^/mei2::ura4^+^ ade6-M210/ade6-M216 leu1/leu1 ura4-D18/ ura4-D18*

JZ409 *h^-^ pat1-114 ade6-M216 leu1*

JZ403 *h^+^/h^-^ ste11::ura4^+^/ste11::ura4^+^ ade6-M210/ade6-M216 leu1/leu1 ura4-D18/ ura4-D18*

JY450 *h^90^ ade6-M216 leu1*

JZ127 *h^90^ mei2::ura4^+^ ade6-M216 leu1 ura4-D18*

JZ396 *h^90^ ste11::ura4^+^ ade6-M216 leu1 ura4-D18*

NJ761  *h^-^ sty1::kan leu1:: P41nmt1-sty1-3Pk<<ura4^+^ ura4-D18*

NJ767  *h^-^ sty1::kan leu1:: P41nmt1-sty1K49R-3Pk<<ura4^+^ ura4-D18*

|  |
| --- |
